# Assessment of the Extirpated Maritimes Walrus Using Morphological and Ancient DNA Analysis

**DOI:** 10.1371/journal.pone.0099569

**Published:** 2014-06-12

**Authors:** Brenna A. McLeod, Timothy R. Frasier, Zoe Lucas

**Affiliations:** 1 Biology Department, Saint Mary's University, Halifax, Nova Scotia, Canada; 2 Nova Scotia Museum of Natural History, Halifax, Nova Scotia, Canada; 3 The Friends of the Green Horse Society, Halifax, Nova Scotia, Canada; State Natural History Museum, Germany

## Abstract

Species biogeography is a result of complex events and factors associated with climate change, ecological interactions, anthropogenic impacts, physical geography, and evolution. To understand the contemporary biogeography of a species, it is necessary to understand its history. Specimens from areas of localized extinction are important, as extirpation of species from these areas may represent the loss of unique adaptations and a distinctive evolutionary trajectory. The walrus (*Odobenus rosmarus*) has a discontinuous circumpolar distribution in the arctic and subarctic that once included the southeastern Canadian Maritimes region. However, exploitation of the Maritimes population during the 16^th^-18^th^ centuries led to extirpation, and the species has not inhabited areas south of 55°N for ∼250 years. We examined genetic and morphological characteristics of specimens from the Maritimes, Atlantic (*O. r. rosmarus*) and Pacific (*O. r. divergens*) populations to test the hypothesis that the first group was distinctive. Analysis of Atlantic and Maritimes specimens indicated that most skull and mandibular measurements were significantly different between the Maritimes and Atlantic groups and discriminant analysis of principal components confirmed them as distinctive groups, with complete isolation of skull features. The Maritimes walrus appear to have been larger animals, with larger and more robust tusks, skulls and mandibles. The mtDNA control region haplotypes identified in Maritimes specimens were unique to the region and a greater average number of nucleotide differences were found between the regions (Atlantic and Maritimes) than within either group. Levels of diversity (*h* and π) were lower in the Maritimes, consistent with other studies of species at range margins. Our data suggest that the Maritimes walrus was a morphologically and genetically distinctive group that was on a different evolutionary path from other walrus found in the north Atlantic.

## Introduction

The biogeography of a species is a result of a complex series of past and current climate changes, ecological interactions, anthropogenic impacts, physical geography, and evolution. To fully understand the contemporary biogeography of a species, and to make projections for future survival, it is necessary to understand the species' history. It is important to examine specimens from regions of localized extinction, as the loss of a species from such regions may represent the loss of unique adaptations and the loss of a potentially distinct evolutionary trajectory for the species. Of particular importance are margins of the species range, which are often identified as areas of increased genetic differentiation and isolation as well as morphological adaptation to ‘new’ habitats, or niche evolution [1].

The walrus (*Odobenus rosmarus*) is a large pinniped with a discontinuous circumpolar distribution in the arctic and subarctic. The species is easily recognizable by its large tusks and robust size, and is the only species within its family, Odobenidae. There are currently three recognized subspecies. The Atlantic walrus (*O. rosmarus rosmarus*, Illiger, 1815) is found throughout the Eastern Canadian Arctic to Franz Josef Land, the Barents and Kara Seas. The Pacific Walrus (*O. rosmarus divergens*, Linnaeus, 1758) is found in the Bering and Chukchi Seas. The third subspecies (*O. rosmarus laptevi*, Chapskii, 1940) is found in the Laptev Sea. However, the distinction of this last group has been debated and recent morphological and molecular data suggest that *O. r. laptevi* be considered synonymous with *O. r. divergens* [2]. Following centuries of extensive exploitation, the current status and population size of each subspecies are poorly known and currently being investigated. Hunting still occurs in most regions (Canada, US, Russia, and Greenland), and the species may be threatened by habitat disturbance, pollution and climate change [3].

Contemporary walrus distribution and diversity are a result of the species shifting its range through time, which has been tightly associated with climate changes over the past 5–8 million years. While *Odobenus* likely originated in the Pacific Ocean, approximately 5–8 million years ago (mya), individuals from the Pacific founded the Atlantic stock through the Panama seaway [4]. Subsequently, the Pacific stock is thought to have become extinct ∼2mya [5] and it is suggested that the current Pacific subspecies originated by recolonization from the Atlantic, through either the Canadian or Russian Arctic during a subsequent interglacial period [6].

In the North Atlantic, fossil walrus specimens identified throughout the eastern North American seaboard (Virginia, Maine, North Carolina, New Jersey) [7] suggest a more southerly distribution during the Wisconsinan Glaciation/early Pleistocene. However, with climate warming following the last glacial maximum (LGM), the Atlantic walrus distribution shifted northward reaching the Bay of Fundy and the Grand Banks by 12,500–12,800 before present (BP), southern Labrador by 11,500 BP, and the central Canadian Arctic by 9,700 BP [8].

**Figure 1:**
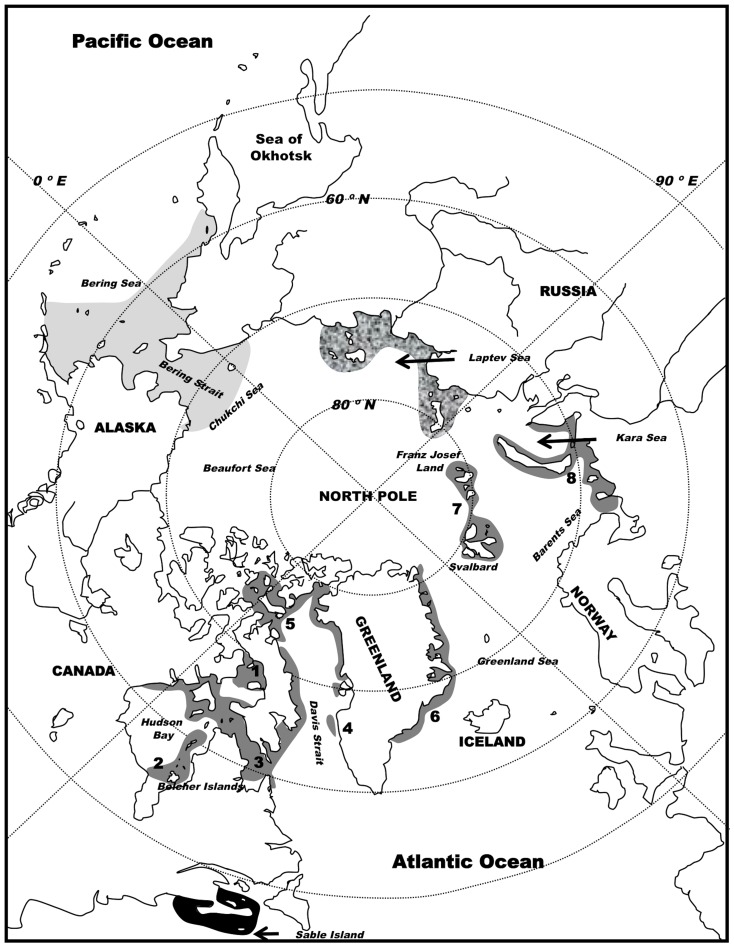
Distribution of the walrus. PAC, ATL, and LAP groups are indicated in light gray, dark grey and mottled gray, respectively. Area of MAR historical specimen sampling for this study is shown in black. Atlantic subpopulations are: (1) Foxe Basin, (2) southern and eastern Hudson Bay, (3) northern Hudson Bay/Hudson Strait/northern Labrador, (4) western Greenland, (5) in the ‘North Water’ (Baffin Bay/northeastern Canadian Arctic, (6) eastern Greenland, (7) Franz Josef Land/Svalbard, and (8) Kara Sea/Barents Sea.

Today ∼8 subpopulations of the Atlantic walrus remain. Five of these are found west of Greenland and three are found east of Greenland [9] (but see [10], [11] for recommendations on further subdivision)(Fig. 1). Support for the recognition of subpopulation status of these groups (e.g. for management purposes) has been found in both morphological and genetic data for most of these groups (e.g. [9], [12], [13], [2]).

The walrus once inhabited the Canadian Maritimes (waters of the Eastern Canadian provinces of Quebec, New Brunswick, Nova Scotia, and Prince Edward Island), with a population suggested to have been greater than 100,000 during the 17^th^ century [14]–[16]. Because it was prized for its ivory-like tusks, thick hide and blubber [7], it was heavily hunted during the 16^th^–18^th^ centuries, particularly around Sable Island, Nova Scotia; the Magdalene Islands; Prince Edward Island; other Gulf of St. Lawrence islands; and off the coast of New Brunswick and Cape Breton [8], [17], [18]. The species is now extirpated from these areas and has not been common south of 55°N for ∼250 years [19], [20]. Although there have been very occasional sightings of apparent strays in the region (e.g. [21], [20], [22], [23]), there have been no signs of recolonization and recovery is considered unfeasible [24].

Some data suggest that the Maritimes walrus was distinct from other Atlantic walrus. Although there are no living examples, numerous postglacial walrus specimens have been identified in New Brunswick, Nova Scotia, Newfoundland, and throughout the Gulf of St. Lawrence [19], [25]–[27]. C. R. Harington and the late F. H. Fay conducted preliminary analyses of 92 adult (72 male, 20 female) postglacial walrus from the Maritimes region. They found that this walrus was larger overall, and had very large upper incisors (I3) relative to the Atlantic walrus (pers. comm. to C. R. Harington, 1992, reported by [8]). They suggested the skull specimens, though different in shape, appeared similar to the Pacific walrus which tends to be ∼3% taller and ∼10% heavier than the Atlantic walrus, and is recognizable by its longer tusks and broader snout [4], [28]. In addition, larger size was evident in large average rostral width and condylobasal length (F. H. Fay pers. comm. to ZL).

We assessed the Maritimes walrus within what is known about global and regional walrus stock structure. The walrus, like several other arctic and subarctic marine mammal species, such as the bowhead (*Balaena mysticetus*) and beluga whales (*Delphinapterus leucas*), appears to have recently inhabited the waters of southeastern Canada. The fact that the area has not been recolonized by walrus may indicate the previous inhabitants were either a distinct, isolated (and perhaps specially adapted) group, or climatic changes over the past 250 years have rendered the habitat now unsuitable. If the group was relatively isolated from more northerly groups, it may have been more genetically and/or morphologically differentiated as well. If this group was adapted for a warmer habitat, its loss represents the loss of evolutionary potential for the species.

For this research, morphological and genetic characteristics of cranial specimens of the extirpated Maritimes walrus collected in Nova Scotia, New Brunswick and Quebec, Canada were compared to those of specimens from current Atlantic and Pacific populations to examine the relationship between the Maritimes group and other walrus subspecies.

## Materials and Methods

### Morphological Analysis

**Figure 2:**
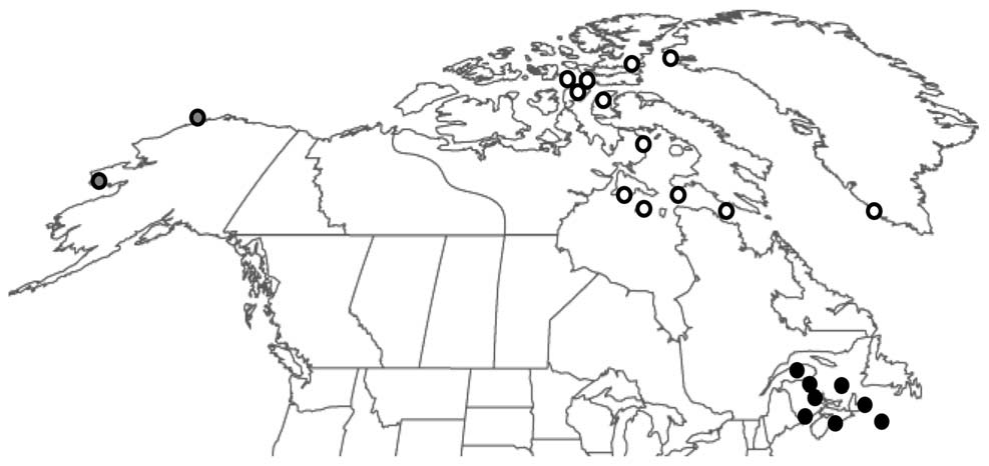
Collection sites for walrus specimens providing morphological data. Black, gray, and white centered circles denote sites of MAR, PAC, and ATL samples respectively. Table S1 gives specific sample origins.

Morphological data were collected from cranial and mandibular bones held in both public and private collections (n = 278). This included 116 Maritimes (MAR)(*Odobenus rosmarus rosmarus*) specimens (65 cranial/51 mandibular elements), 156 specimens from the Eastern Canadian Arctic (the Atlantic subspecies, ATL) (*Odobenus r. rosmarus*)(82 cranial/74 mandibular elements), and 6 from the North Pacific (the Pacific subspecies, PAC) (*O. r. divergens*) (4 cranial/2 mandibular elements) (Table 1, Fig. 2, Table S1). Most specimens from the ATL have tusk length-at-age and sex information (after [29]). The cranial sample includes 41 males and 30 females while the mandibular sample contains 34 males and 27 females. Ages, as determined by Mansfield [29] ranged from 2.1 – 26+ years. The authors confirm that access to these specimens can be granted to other researchers upon request.

**Table 1:** Number of cranial and mandibular samples for which morphological data were collected from public and private institutions/collections.

Specimen type/Region	Collection				
	*NSMNH*	*NBM*	*CMN*	*Private*	Total
Mandibular bone(s) – ATL	0	0	74	0	74
Cranial bones – ATL	0	0	82	0	82
Mandibular bone(s) – MAR	32	5	10	4	51
Cranial bones – MAR	38	4	7	16	65
Mandibular bone(s) – PAC	0	0	2	0	2
Cranial bones - PAC	0	0	4	0	4
**Total**	**70**	**9**	**179**	**20**	**278**

‘NSMNH’, ‘NBM’, and ‘CMN’ denote Nova Scotia Museum of Natural History, New Brunswick Museum, and Canadian Museum of Nature, respectively.

**Figure 3:**
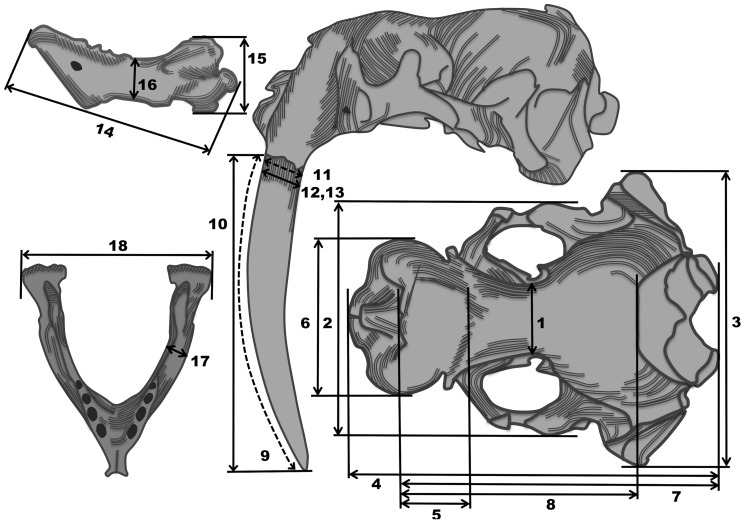
Cranial, tusk and mandibular measurements collected from walrus crania where possible. Numbers indicate measures taken as described in Table 2. Dashed lines indicate measures taken with a measuring tape. Measure 13 is not shown but is a mediolateral equivalent of measure 12. Images modified from Allen [7].

Morphological data were collected using measures previously outlined for seals by the Committee on Marine Mammals [30] and those used previously in *Odobenidae* (after [31], [32], and [2]). Additional measures were used that 1) are commonly used in assessing mammalian cranial morphology; 2) served to capture additional information from skulls in cases where parts of the skull were missing due to fracture or wear (e.g. occipital condyles) and 3) added additional morphological information that we thought was appropriate and informative. Where specimen condition allowed, 18 metric characteristics were examined (Table 2; Fig. 3). These included 5 mandibular measures and 13 cranial measures. Measurements greater than 30.48 cm were taken using vernier calipers (to the nearest 1.0 mm). Measurements less than 30.48 cm were taken using digital calipers (Mitotoyu) (to the nearest 0.01 mm). Tusk circumference and curvilinear tusk length were measured using a flexible measuring tape (to the nearest 1.0 mm). All measurements were taken by one of the authors (BAM).

**Table 2:** Morphological measurements taken and corresponding definitions.

Measure	Name	Definition
***Cranial Measures***		
1	*Interorbital width (IW)* *	least distance between the orbital fossae
2	*Zygomatic width (ZW)* *	greatest width, at right angle to the axis of the skull, across the zygomatic arches
3	*Cranial width/mastoid width (CW)* *	greatest transverse width of the skull posterior to the zygomatic arches
4	*Condylobasal length (CBL)* *	measured from a transverse line touching the most posterior points on the occipital condyles to a transverse line touching the most anterior points on the premaxillary bones
5	*Nasal length (NL)*	greatest length from the anteriormost to posteriormost points of the nasal bones
6	*Rostral width (RW)* *	greatest width of the maxillary bones at the level of the canines
7	*Occiptonasal length (ONL)*	greatest length from the tip of the nasals to the posterior of the occipital condyles
8	*Nasal-occipital crest (N-Oc)*	measured from the most anterior tip of the nasals to the most anterior tip of the occipital crest
9	Curvilinear tusk length (TL-C)***	curvilinear length from alveolar margin to the tip along the anterior edge of the tusk
10	Straight tusk length (TL-S)***	straight line length from the outer alveolar margin to the tusk tip
11	Tusk circumference (TC)***	tusk circumference at the alveolar margin
12	*Tusk diameter (anterposterior) (TD-AP)*	maximum anterposterior diameter at alveolar margin
13	*Tusk diameter (mediolateral) (TD-ML)*	maximum mediolateral diameter at alveolar margin
***Mandibular Measures***		
14	Mandible length (ML)**	distance between most anterior point on mandible and midpoint on posterior surface of articular condyle
15	Mandible height (MH)**	distance between most dorsal point on coronoid process and most ventral point on angular process
16	Least mandible depth (MD)**	minimal distance between dorsal and ventral mandibular surfaces, posterior to the last post-canine
17	Least mandible thickness (MT)**	minimal lateral distance between medial and lateral mandibular surfaces, posterior to the last post-canine
18	*Mandible width (MW)*	maximum mediolateral distance between the left and right mandibular condyles

*After Committee on Marine Mammals (1967), **Wiig and Gjertz (1996) and ***Wiig et al. (2007).

### Morphological Analysis – Data Standardization and Organization

For several of the mandibular and tusk measures, both left and right sides were examined (measures #9–17). Prior to analysis, we examined whether there was any lateral asymmetry by regressing measures from one side on the other and examining the slope of the regression line as well as whether its standard error encompassed 1.0. Lateral asymmetry was identified in most of the measurements, therefore analyses were carried out using the side with the most data available. To quantify the amount of average individual percent directional asymmetry (DA) present we used the formula %*DA  =  (Right - Left)/(Average of Right and Left)×100* (e.g. [33]–[35]). This measure allowed us to directly compare the amount of DA present irrespective of dimensions of very different sizes. We also calculated percent absolute asymmetry (%AA) (e.g. [33]); %AA  =  (maximum value – minimum value)/(average of the two values)×100. This measure allowed us to examine the amount of asymmetry present regardless of directionality, or the amount of “random” asymmetry. To examine whether the %DA and %AA were significantly different between regions, we used student's t-tests with a Bonferroni correction for multiple tests [36].

**Figure 4:**
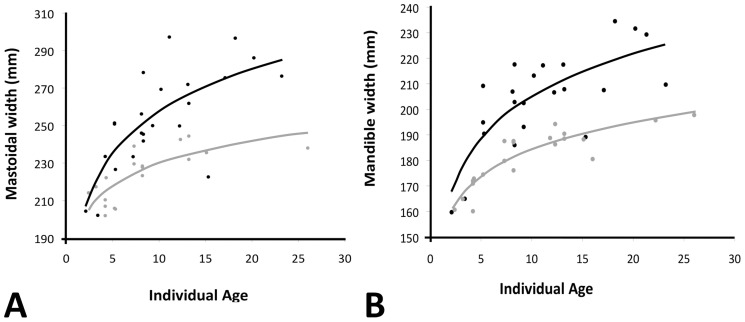
Mastoid width (a) and mandible width (b) versus age in known age males (black) and females (gray) from ATL.

The goal of this research was to determine if there are differences between animals of ATL and MAR populations; however, such a comparison may be difficult when the sample set is comprised of individuals of varied ages and sexes. Therefore, all measures were standardized by a morphological measure that has a relationship with individual age and sex. To determine an appropriate measure for this standardization, we examined the ATL specimens of known age and sex. We found that mastoidal width (#3) and mandible width (#18) were appropriate measures because they showed the strongest relationship with sex and age (Fig. 4). To standardize the analyses, each mandibular measure (#14–#17) was divided by that specimens corresponding mandible width, and all skull and tusk measures (#1, #2, #4–13) were divided by corresponding mastoid widths. Standardized values were then used in all further analyses.

### Morphological Analysis - Tests of normalcy, equal variance, and equal means across regions

We evaluated whether the data collected were normally distributed and whether the variances were equal using the Shapiro-Wilk normality test [37] and Fisher's F Test. The means of variables that were normally distributed with equal variances were compared using a Welch 2-sample t-test [38], while all other means were compared using a non-parametric Wilcoxon rank-sum test [39]. These analyses were conducted using only ATL and MAR samples, as PAC sample sizes were too small (n = 1–4, depending on measure evaluated). All tests were conducted using the program R [40].

### Discriminant Function Analysis of Principal Components

Standardized skull, mandible, and tusk data were used to conduct a Principal Component Analysis (PCA) in R using the MASS library [41]. The resulting principal components were then used to carry out discriminant function analysis (DFA) with the ADE4 package [42]. Prior to this analysis, specimens that were missing any data/measures were removed. Using PCs in a DFA allows us to examine differentiation between the regions, while decreasing the total number of variables into a small number of principal components or ‘factors’ that account for most of the variance present in the datasets. In this way, we can then examine which factors/variables explain most of the differences between the datasets. We are interested in the differences in skulls from geographic regions, with measures as a proxy for skull shape and size. PCA captures uncorrelated patterns of skull shape and size that could be more informative than the measures themselves. Conducting PCA prior to DFA serves to transform data into uncorrelated variables, which is a requirement for DFA. DFA then allows us to compare the groups. Discriminant analysis of principal components (DAPC) has been shown previously to be a highly effective and relatively rapid method for assessing differentiations between groupings (e.g. [43], [44].

### Genetic Analysis

Bone shavings were collected from historical specimens using a cordless drill following the sampling procedure of McLeod et al. [45]. This procedure minimizes DNA contamination and maximizes specimen preservation and integrity. In addition, tissue specimens (tongue and tendon) and DNA from contemporary walrus populations from the Eastern Canadian Arctic were provided by the Makivik Corporation and the Department of Fisheries and Oceans (DFO), respectively. DNA provided from DFO was extracted previously using the Sigma DNA extraction kit or Qiagen DNEasy tissue kit (Qiagen). Contemporary samples were originally collected during aboriginal hunts (Makivik Corporation), or through biopsy sampling (DFO).

**Figure 5:**
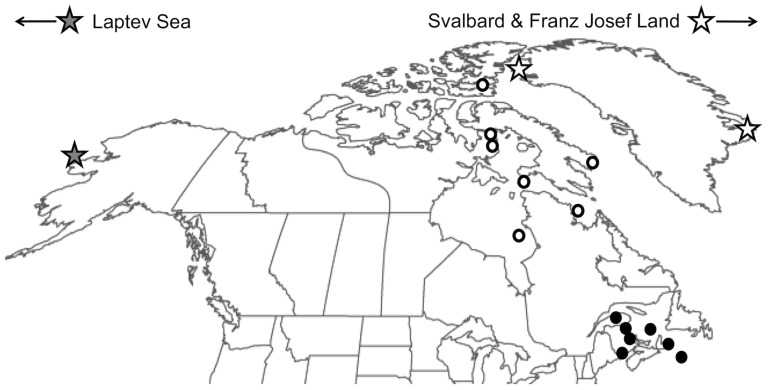
Collection sites for walrus specimens providing DNA data. Black, gray, and white centered shapes denote MAR, PAC, and ATL regions respectively. Circles indicate new data collected in this study, stars indicate data presented previously [2]. Not shown on the map are samples from Laptev Sea, Svalbard and Franz Josef Land of Lindqvist et al. [2].

A total of 125 samples were collected for DNA analysis (Table S2, Fig. 5) including 37 from the Maritimes (public and private collections) and 88 comparative samples from the Eastern Canadian Arctic. The latter included 74 samples of DNA from southeast Baffin Island (DFO) and 14 tissue samples of contemporary walrus from Nunavik (Akpatok Island in Ungava Bay; Salisbury Island and Notthingham Islands in Hudson Strait, and Sleepers Island in Eastern Hudson Bay)(Makivik Corporation).

DNA was extracted from contemporary specimens using the DNEasy blood and tissue kit (Qiagen) then quantified using a NanoDrop 2000 spectrophotometer (Thermo Scientific Inc.). DNA was extracted from historical specimens using the protocols of Rastogi et al. [46] following stringent ancient DNA (aDNA) protocols to prevent contamination of materials (as per [45] and [47]). All pre-polymerase chain reaction (PCR) aDNA work was conducted in a separate room and in isolation from any contemporary specimens or materials that have been used in association with contemporary materials. In addition, samples were extracted in small batches (n = 5–8) as a means to minimize cross-contamination. Blank samples (samples not containing DNA) were included in each step of the contemporary and historical DNA analyses as a means to identify contamination.

It has been previously shown that the different Atlantic subpopulations of walrus can be distinguished based on analysis of mitochondrial DNA (e.g. [2], [9]). Therefore, we PCR amplified all 125 samples for the mitochondrial control region using the primers DL-2F and DL-3R of Lindqvist et al. [2] which amplify a region that is approximately 428 bp. For contemporary DNA samples, PCR cycling conditions consisted of an initial five-minute denaturation step at 94°C; 30 cycles of 94°C for 30 seconds, 57°C for one minute, and 72°C for one minute; and a final extension step at 60°C for 45 minutes. PCR cocktail conditions were as follows within a 15 µl reaction: 10 ng DNA, 1X PCR buffer (20 mM Tris-HCl pH 8.4, 50 mM KCl) (Invitrogen, Burlington, ON), 1.5 mM MgCl2 (Invitrogen, Burlington, ON), 0.2 mM each dNTP (Amersham Biosciences, Piscataway, NJ), 0.3 µg/µl BSA (Sigma, Oakville, ON), 0.05 U/vl Taq polymerase (Invitrogen, Burlington, ON), and 0.3 µM of each primer. For historic DNA samples, conditions were identical with the exception of a 20 µl reaction volume, 2 µl DNA (of unknown concentration), and 50 PCR cycles. To examine amplification success and DNA quantity, amplified DNA was visualized under UV light following electrophoresis through 1.5% agarose gels stained with ethidium bromide.

To prepare DNA sequences for analysis, dNTPs and excess primers were first degraded. We incubated samples for 15 minutes at 37°C, then 15 minutes at 80°C within a 5.78 µl cocktail containing 5 µl amplified DNA, 1.29X Antarctic phosphatase buffer (50 mM Bis-Tris-Propane-HCl, 1 mM MgCl2, 0.1 mM ZnCl2, pH 6.0), 0.1 U/µl Antarctic phosphatase (New England Biolabs), and 0.123 U/µl exonuclease I. Samples were then sequenced using an ABI BigDye Terminator v3.1 Cycle Sequencing Kit (Applied Biosystems), de-salted using ethanol precipitation [48], and size separated and visualized using capillary electrophoresis on an ABI 3500xl Genetic Analyzer (Applied Biosystems). All MAR samples were sequenced using both primers. Sequences were then examined visually using 4Peaks [49] and edited in BioEdit ver. 7.1.3 [50]. A multiple sequence alignment was carried out using ClustalX ver. 2.1 [51]. All historical samples were sequenced in both directions (using each primer), while contemporary samples were sequenced in one direction only. Unique mitochondrial control region haplotypes were then determined by eye and assigned to samples. Any samples with unique haplotypes (found only once within the sample set) were reamplified and sequenced for confirmation.

Haplotypes from this study were then compared to those available from contemporary populations in the Pacific, Laptev Sea and Atlantic regions by Lindqvist et al. [2]. As first steps towards evaluating the amount of evolutionary divergence both within and between groups, we examined nucleotide diversity (π) and haplotype diversity (*h*) using DnaSP ver. 5.10.01 [52]. To visualize relationships between individual haplotypes, a median joining network was constructed using the program Network 4.610 [53]. We used the HKY [54] model of molecular evolution with gamma distributed rate variation across sites and a proportion of invariant sites (HKY+G+I), as indicated by MODELGENERATOR to be appropriate for our data set [55] to construct a phylogenetic tree using Bayesian inference of phylogeny approach implemented in MrBayes ver. 3.2 [56]. Analysis incorporated running 4 chains for 5,000,000 generations, with a sample taken every 500 generations, and 1,250,000 (25%) initial steps discarded as burn-in. This tree was visualized using FigTree ver. 1.3.1 [57].

## Results

### Morphological Analysis

**Figure 6:**
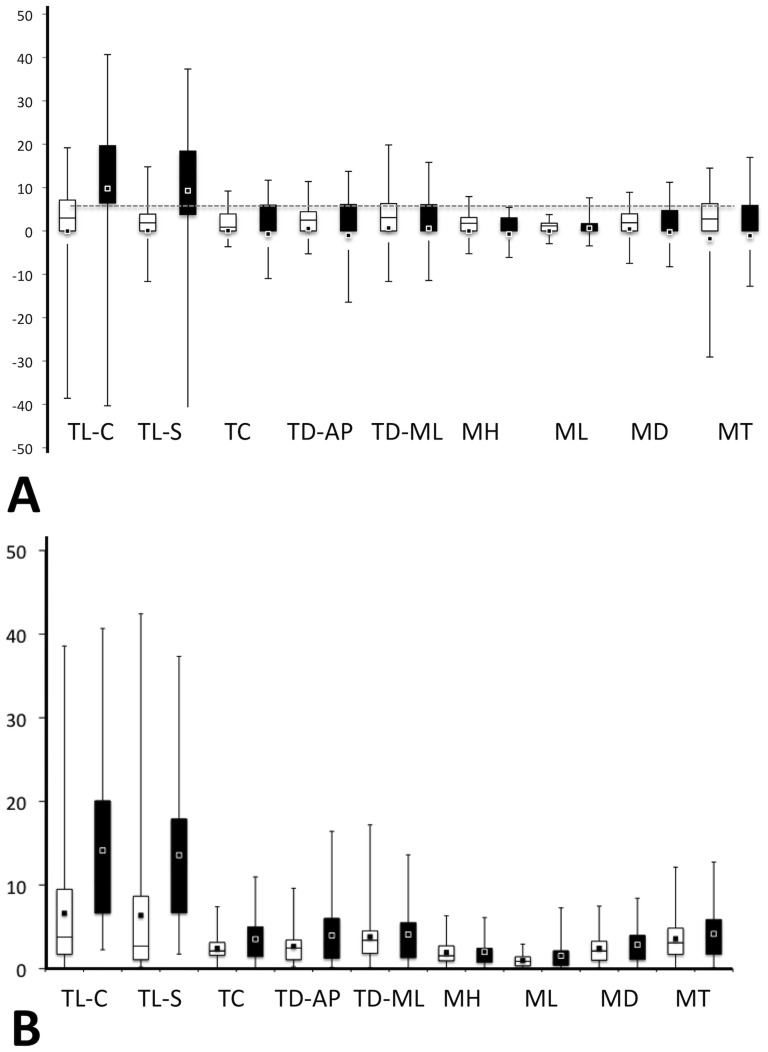
Boxplots of (a) %DA and (b) %AA. Whiskers denote minimum and maximum of the data. Boxes denote the 2^nd^ and 3^rd^ quartiles of the data and the squares denote the data mean. For %DA, negative values indicate a left-side asymmetry while positive values indicate a right-side asymmetry.

Regression analysis suggested that lateral asymmetry was evident in 6 of the 9 tusk and mandible measures for which left and right sides were examined. Both ATL and MAR showed lateral asymmetry for all tusk measures. While MAR did not show signs of asymmetry in mandibular measures, ATL had slight asymmetry for mandible thickness. Because of this asymmetry, right sides were not exchanged for missing left sides when standardizing the datasets. As a result, the sides with a higher count (left sides for all measures) were used. To further examine asymmetry in the specimens, we also examined %DA and %AA separately for each of the regions (Table 3; Fig. 6) and whether average %DA and %AA within each region was significantly different. After incorporating a Bonferroni correction, only two measures were significantly different between the regions: straight and curvilinear tusk length (Measures #9 and #10). We found that MAR average individual %DA was ∼10%, with right tusks longer than left. For the remaining measures, %DA ranged from 0.017%–1.79% while %AA ranged from ∼0.978%–4.181% with the MAR exhibiting greater %AA for all measures.

**Table 3:** Regional average percent individual directional asymmetry (%DA) and percent individual absolute asymmetry (%AA) in the MAR and ATL specimens.

Measure:	%DA		Significance	%AA		Significance
	MAR	ATL		MAR	ATL	
**TL-C (#9)**	**9.781**	**−0.033**	**t = 3.8403, p = 0.0003**	**14.63**	**5.628**	**t = 3.9893, p = 0.0002**
**TL-S (#10)**	**9.278**	**0.084**	**t = 3.0414, p = 0.0036**	**15.07**	**3.912**	**t = 5.64, p = 0.0001**
TC (#11)	−0.699	0.068	t = 0.8450, p = 0.4013	3.591	2.363	t = 2.3530, p = 0.0218
TD-AP (#12)	−1.026	0.559	t = 0.8421, p = 0.4028	3.977	2.671	t = 1.8982, p = 0.0620
TD-ML (#13)	0.568	0.697	t = 0.1002, p = 0.9205	4.181	3.732	t = 0.5295, p = 0.5983
ML (#14)	0.617	−0.017	t = 1.7243, p = 0.0882	1.544	0.978	t = 2.2098, p = 0.0297
MH (#15)	−0.733	−0.095	t = 1.133, p = 0.2603	2.003	1.616	t = 1.2916, p = 0.1999
MD (#16)	−0.261	0.462	t = 1.0622, p = 0.2907	2.854	2.451	t = 0.9785, p = 0.3302
MT (#17)	−1.09	−1.79	t = 0.6751, p = 0.5010	4.165	3.962	t = 0.2809, p = 0.7794

**Figure 7:**
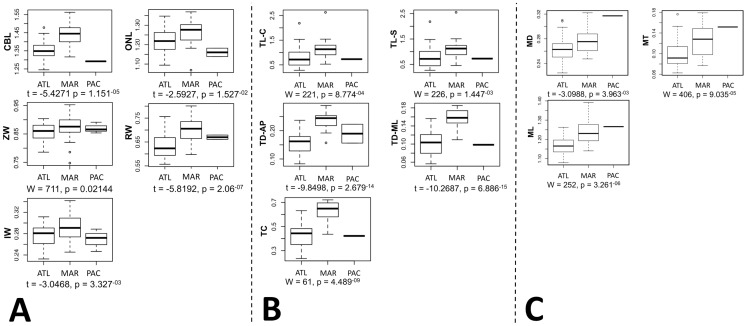
Boxplots for standardized measures of (a) skull, (b) tusk, and (c) mandible data with corresponding *t* or *W* values for the comparison of ATL and MAR regions. Measurement abbreviations correspond to those described in Table 2.

Parametric and non-parametric tests of means of each of the morphological variables showed that 13 of the 16 comparisons were significantly different (p<0.05) between the ATL and MAR groups (Fig. 7). Skull measurements corresponding to overall skull length (CBL, ONL) and width (RW, IW) were significantly different across regions, with MAR longer and wider than ATL, while other measures (NL, N-Oc) were not. Mandible length, depth and thickness were significantly different between the two regions, with MAR values being greater, while mandible height was not significantly different. Finally, all tusk measures were significantly different between the regions, with MAR tusks longer and more robust than ATL tusks. Although four PAC specimens were included in the boxplots (Fig. 7) the sample size is too small for these data to be used in any interpretation of the relationships between these regional groupings. It should be noted again that all these results are based on the standardized data, and not original data, therefore we have accounted for sex and age differences in the datasets.

**Figure 8:**
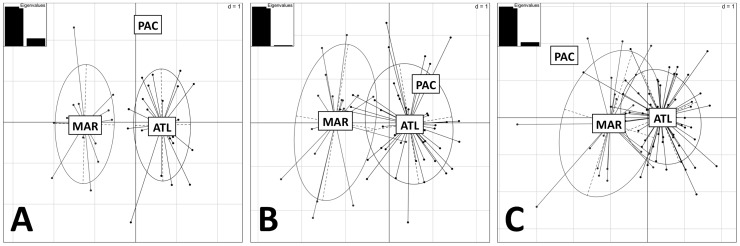
Discriminant function analysis scatterplots for skull (a), tusk (b), and mandible (c) data. Embedded is also a plot of the first two eigenvalues of the discriminant analysis. Circles encompass the range of 2/3 of the data points.

We conducted discriminant function analysis (DFA) using the principal components (PCs) of the PCA. For the skull data, the first two (of seven) PCs accounted for 73.7% of the variance in the dataset. For the mandible data, the first two PCs (of four) accounted for 75.2% of the variance, and for the tusk data the first two (of five) accounted for 99.2% of the variance. In Fig. 8, the DFA scatterplots from each of the three regions are plotted for the skull, mandible and tusk data. In all three graphs there is differentiation between the ATL and MAR regions, and sometimes the PAC. Although differentiation is evident, particularly between tusk and skull measures, there is still considerable overlap in the mandible and tusk distributions. However, there was complete isolation of the ATL and MAR groups within the DFA analysis of skull characteristics.

### DNA Extraction, Amplification, Sequencing and Phylogenetic Analysis

Mitochondrial DNA was successfully amplified and sequenced from all 88 of the extracted contemporary specimens and 28 of the 37 extracted historic specimens. We obtained clean and readable sequences that were 344 bp in length. The final haplotype length was largely determined by the sequence length obtained from contemporary specimens, since they were sequenced in a single direction. Among these sequences, 28 unique haplotypes were identified within the contemporary ATL (Genbank accession KJ522887 - KJ522911, KJ522920 - KJ522922) and 8 within the historic MAR specimens (Genbank accession KJ522912 – KJ522919). Haplotypes from this study were then compared to those available from Lindqvist et al. [2]; however, because our sequences were shorter than those of Lindqvist et al. [2], three of their haplotypes were collapsed into one (Sequence IDs ATL13/ATL14, a unique haplotype, became identical to ATL11 and ATL21, two other haplotypes). Across the two studies, 3 of our ATL haplotypes were shared with the Lindqvist et al. [2] Atlantic specimens. These were the same as the following sequence IDs from Thule, NW Greenland: ATL07/ATL17, ATL09/ATL10/ATL16, and ATL6/ATL08. However, no haplotypes were shared between historic MAR specimens and any other Atlantic specimens.

Haplotype diversity (*h*), nucleotide diversity (π), and the average number of nucleotide differences both within and between regions were calculated for samples from PAC, Laptev Sea (LAP), ATL, and MAR (Table 4). Both *h* and π were found to be the lowest in the MAR, while they were the highest in the PAC. In addition the MAR had the lowest number of nucleotide differences between sequences.

**Table 4:** Haplotype (*h*) and nucleotide diversity (π) and corresponding standard error (in parentheses) for PAC, LAP, ATL and MAR regions.

Region	PAC	ATL	MAR	LAP
	*h* = 0.967 (0.036)	*h* = 0.844 (0.024)	*h* = 0.439 (0.114)	*h* = 0.800 (0.164)
	π = 0.02574 (0.00340)	π = 0.00666 (0.00031)	π = 0.00332 (0.00121)	π = 0.00468 (0.00096)
**PAC**	8.675			
**ATL**	13.980	2.230		
**MAR**	14.469	4.644	1.119	
**LAP**	7.463	12.957	13.850	1.600

Below this is the average number of nucleotide differences within (on diagonal) and between regions.

**Figure 9:**
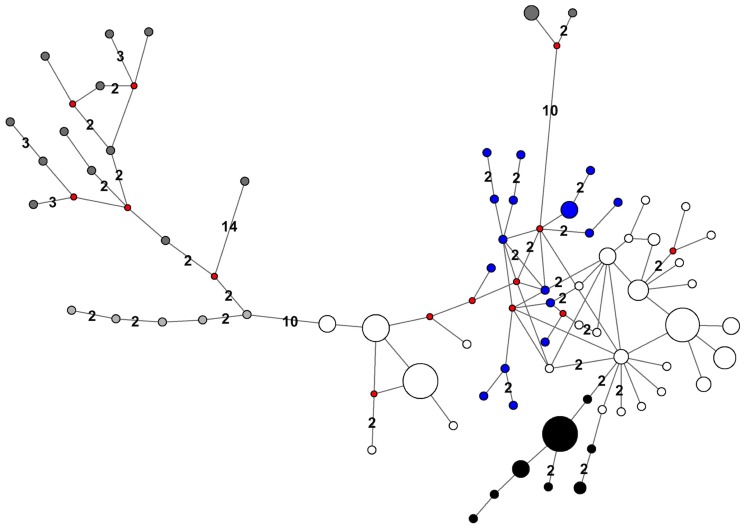
Median joining network of walrus haplotypes from PAC (dark gray), LAP (light gray), ATL (white) and MAR (black) regions as found in Lindqvist et al. [2] and this study. In addition, ATL samples from east of Greenland are indicated in blue. Each line segment denotes one sequence difference, except where numbers indicate differences between more divergent sequences. The circle sizes are indicative of haplotype frequencies across the two studies. Small red circles indicate inferred median vectors.

Haplotypes from PAC, LAP, and ATL (both east and west of Greenland) appear to cluster together within the network, with the exception of two PAC haplotypes that fall out in the network closer to ATL haplotypes from east of Greenland (Fig. 9). Although the samples from the MAR did not share any haplotypes with the PAC/LAP group or the ATL group, the MAR sequences are more similar to the ATL than the PAC/LAP specimens. Many of the ATL haplotypes are highly similar, with most differing from each other by only a single basepair. In addition, the MAR haplotypes appear to branch off in two separate lineages from a single ATL haplotype. Haplotypes that are most similar (and closest) to the MAR haplotypes within the network originate from animals sampled around Southeast Baffin Island and Nottingham Island.

**Figure 10:**
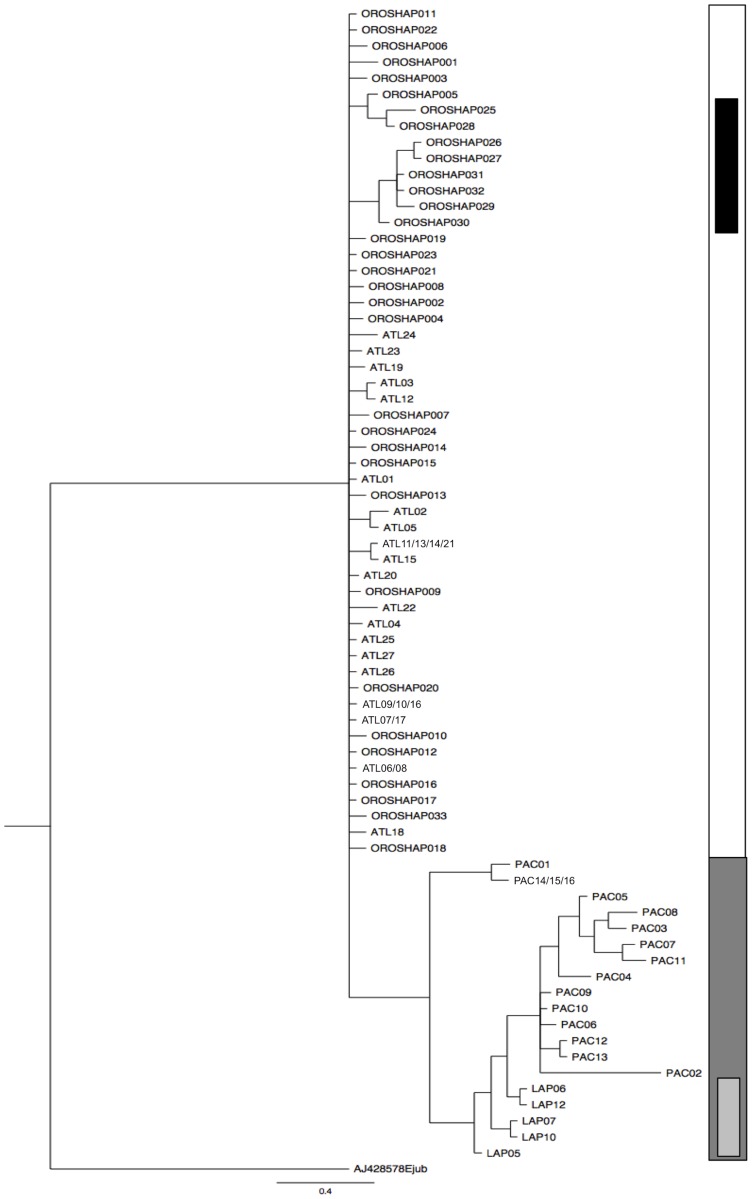
Phylogenetic tree of mtDNA control region haplotypes identified in this study and by Lindqvist et al. [2]. Samples from LAP, PAC, ATL and MAR are shown highlighted by light gray, dark gray, white, and black, respectively. The Stellar sea lion (*Eumetopias jubatus*) is used as an outgroup.

Similar to the median joining network, the phylogenetic tree shows the ATL and PAC/LAP subspecies grouping within separate and distinct clades, with the MAR specimens grouping within the ATL clade (Fig. 10).

## Discussion

The morphological analyses presented here indicate that the Maritimes walrus was a physically distinctive group from other populations of walrus in the North Atlantic, west of Greenland. Comparisons of morphological measures across ATL and MAR indicated that most of the cranial and mandibular measures were significantly different between the two groups, with MAR values being greater in all cases. The discriminant analysis of principal components (DAPC) also identified the ATL and MAR as distinctive groups, with complete isolation of skull features. The MAR group appears to have been comprised of larger animals, with larger and more robust tusks, skulls and mandibles. This agrees with previous suggestions that the group was morphologically distinct, and more robust in overall size (pers. comm. to CRH, 1992, reported by [8]). More thorough morphological comparison of the MAR and PAC groups is required.

Previous studies of marine mammals have identified morphological variation in relation to environmental characteristics such as latitude, average water temperature (*Tursiops* spp.) [58], and primary productivity (*Phocoenoides dalli*) [59]. It is not known what selective pressures may have resulted in the differences in size that have been identified here. Morphological differences identified in the MAR group may be a result of genetic divergence and/or environmental effects on phenotype. At this southerly habitat, at the margin of historical walrus distribution, MAR individuals may have been subjected to warmer conditions, as well as different prey and substrate types from those of other walrus populations.

We cannot exclude the possibility that the ATL walrus is less robust than the extirpated MAR walrus as a result of selective hunting pressures towards larger, more robust animals in the Canadian Arctic. The walrus has a long history of exploitation, both aboriginal and commercial. Prized for its blubber and ivory, larger individuals (with larger tusks) were likely preferentially hunted. If exploitation was extensive and skewed towards the hunt of more robust individuals with larger tusks this may have resulted in a phenotypic shift towards smaller individuals with smaller tusks (e.g. [60]).

The DNA analysis indicates that the extirpated MAR group was most genetically similar to contemporary ATL population(s). The MAR haplotypes identified are not monophyletic, as would be expected following long isolation and subsequent genetic divergence, and instead fall within a larger clade including ATL haplotypes. However, in support of the hypothesis that the MAR group was distinctive, we found no shared haplotypes between the regions. All haplotypes identified within the MAR are located within two distinctive and closely related lineages. As well, there are a greater average number of nucleotide differences between the ATL and MAR than within either group suggesting some degree of divergence. Finally, levels of diversity (h and π) were lower in the MAR. This finding is consistent with many previous studies indicating lower levels of genetic variation at range edges, especially in areas where greater isolation has occurred; these include arctic marine mammals (e.g. beluga, bowhead, Saimaa ringed seal (*Pusa hispida saimensis*) (reviewed by [61])). Unfortunately, we are currently limited in the types of additional analyses that can be conducted (e.g. mismatch distribution, etc.) to estimate a divergence time of the MAR and ATL groups because we do not have radiocarbon ages for the MAR specimens. It is therefore assumed that the samples represent a variety of ages, which could range from ∼250–12,800 BP [8], [27], [62].

Samples from the ATL and PAC exhibit strikingly different haplotype divergence patterns (Figure 10). While haplotypes with the PAC clade are relatively divergent from each other, with long branches, the ATL clade has very short comb-like branching. In addition, ATL exhibits lower levels of haplotype and nucleotide diversity (Table 4). This may suggest a shorter evolutionary history for the ATL clade and is consistent with more recent colonization of the area [6]. The similarity of ATL haplotypes (most branching from a single limb) may be indicative of a population bottleneck event, perhaps resulting from a relatively small number of founding individuals. The fact that the MAR haplotypes exist within this clade suggests that the two groups originated from the same stock of founding individuals, which likely shared southerly habitat during the LGM [7], [8].

The duration and extent to which the MAR region was inhabited by walrus is not known. Prior to ∼12,500–12,880 BP walrus in the North Atlantic inhabited their LGM refugium; south of the Bay of Fundy [8]. By 9,700 BP the species had entered the Central Canadian Arctic [8]. Thus, occupation of the MAR region by walrus was feasible between <12,880 BP ∼1750 [8], [19], [20]. It is not known whether occupation of the coast of southeastern Canada was continual over this ∼11,000 yr period. Radiocarbon dates for walrus specimens found around southeastern Canada are scattered throughout the period [8], [27], [63], [64], with a cluster of bones that date to ∼9,000–10,000 BP [27]. Historical records suggest that the walrus inhabited the area in large numbers between the late 16^th^ century and mid-18^th^ century [7], [14], [15], [19], [20] at which time they were extirpated. It is also not known whether the animals were seasonal or year-round inhabitants. Though highly gregarious, walrus are separated by sex and age classes for most of the year [65] and sites may have been used by different sex or age groups at various times of the year. In historical times, walrus were (at least) present in the spring to give birth on land [7]. Despite the aforementioned uncertainties, our results suggest that individuals inhabiting the area were isolated enough from northerly groups to allow for the accumulation of both morphological and genetic adaptations.

Sable Island has provided an abundance of MAR walrus bones (>190), making it the most productive site. While such a large sample set may partially be a result of dedicated survey efforts (by ZL), it also appears that Sable Island and its coastal waters hosted significantly more walrus relative to other sites. Did Sable Island represent a refugium haulout site during glacial periods? Sable Island is an emerged part of a ∼30,000 km^2^ sand and gravel bank (Sable Island Bank) that was deposited by a Wisconsinan ice front. Lower postglacial sea levels in Atlantic Canada resulted in the emergence of several large banks between 12,000–6000 BP [66]. It is possible that during this time period exposed banks (e.g. Sable Island Bank, Georges Bank, and Grand Bank) provided ideal walrus habitat: haul out sites surrounded by large areas of shallow waters and substrates appropriate to house large communities of bivalve mollusks, the preferred prey of walrus (e.g. *Mya* sp., *Hiatella* sp., and *Serripes* sp.) [67], [68]. Certainly, by the 16^th^ century, when most of Sable Island was again submerged, it, and other islands in Eastern Canada continued to provide adequate habitat for walrus.

We identified directional asymmetry in several measures. In most cases the degree of asymmetry appeared to be small, with no significant difference between ATL and MAR regions. The only significant difference in degree of asymmetry (both %AA and %DA) was tusk length, with right tusks ∼9–10% longer than left tusks in the MAR. We speculate that the directional asymmetry (DA) may indicate lateralized side use (a ‘handedness’), during feeding. This phenomenon has been shown in several marine mammal species (e.g. walrus [69], fin whale (*Balaenoptera physalus*) [70], humpback whales (*Megaptera novaeangliae*) [71], gray whales (*Eschrichtius robustus*) [72]). In some cases, lateralized side use is hypothesized as a cause for morphological asymmetry (e.g. walrus [69], harbour porpoise (*Phocoena phocoena*) [73], gray whales [72]). Although walrus have several feeding methods, one method is to beat one flipper to remove sediments while suction feeding with the muzzle along the sea floor. Levermann et al. [69] found that walrus in northeastern Greenland have a significant preference for beating the right flipper while feeding. The dimensions of right forelimbs (humerus, scapula, ulna) were significantly longer than those on the left, suggesting the animals in that region have a tendency of dextrality [‘right handedness’] in feeding [69]. Within our specimens, it is possible that if animals exhibited a preference for beating with a particular flipper they also had a tilt in body axis while feeding that applied additional pressure and/or abrasion to the left side, which slowed growth and/or abraded the length (of tusks). Indeed, many of the tusks from the MAR region showed extensive wear on the anterior anterior/distal edges, presumably a result of abrasion during feeding. Interestingly, while the MAR specimens show this ‘skew’, the same relationship is not evident in the ATL specimens. Curvilinear tusk length of the right tusk was on average 35 mm (DA = 9.78%) longer than the left for the MAR specimens, while for the ATL specimens the right tusk was on average 2.2 mm (DA = 1.1%) longer than the right. This difference across the two regions may thus reflect different feeding strategies and/or environments in the two regions.

We assumed that our standardization of measures using variables associated with age and sex have minimized any as-yet unidentified age-based affects from differences in the ATL and MAR sample sets. Yet, it is noteworthy that there appeared to be age-dependent differences between the ATL and MAR samples. Many of the ATL individuals were estimated to range from ∼2–26+ years old. The majority of these specimens, despite being from mature animals, still had several unfused cranial sutures, which are known to close in a sequential pattern with age. In contrast, most samples from the MAR were heavily ossified and had few open sutures. This likely suggests that the MAR specimens were much older and more physically mature animals than most from the ATL samples. This apparent skew towards older specimens from the MAR may be a result of 1) the older skulls, that were bound more strongly by sutures and have denser bone, making them more resistant to breakage and erosion; and/or 2) some as yet unknown behavioural/ecological characteristics of the population that inhabited Sable Island (e.g. habitat that was used by older individuals). In addition, it is likely that the modern ATL population from which the bone specimens were taken may have had older individuals selectively removed.

Although questions remain regarding the duration and extent to which the MAR region was inhabited by walrus, our data suggest that the walrus that once inhabited the region was morphologically and genetically distinctive. The MAR group appears to have accumulated unique genetic mutations and was morphologically distinct, suggesting that it was on a different evolutionary path than other walrus found in the north Atlantic. The loss of this group following extensive commercial exploitation represents the loss of adaptive potential of the species.

## Supporting Information

Table S1 **Table of walrus (Odobenus) bone sample specimen information including: sample ID at corresponding institution, origin, bone type as well as radiocarbon date, sex, and age (if known).** Museum abbreviations are as follows: Canadian Museum of Nature (CMN), New Brunswick Museum (NBM), New Brunswick Museum (geology collection) (NBMG), NSM (Nova Scotia Museum), PRIV (private collection).
(XLSX)

Table S2 **Table of samples included in DNA analysis and corresponding results including DNA lab ID, sample ID at corresponding institution, site of origin, tissue type, and mtDNA haplotype.** Museum abbreviations are as follows: Department of Fisheries and Oceans (DFO), Makivik Corporation (MAK), New Brunswick Museum (NBM), New Brunswick Museum (geology collection) (NBMG), NSM (Nova Scotia Museum), PRIV (private collection).
(XLSX)
